# Construction of siRNA/miRNA expression vectors based on a one-step PCR process

**DOI:** 10.1186/1472-6750-9-53

**Published:** 2009-06-02

**Authors:** Jun Xu, Jie Qiong Zeng, Gang Wan, Gui Bin Hu, Hong Yan, Li Xin Ma

**Affiliations:** 1 Institute of Molecular Biology, Biology Faculty of Hubei University, Wuhan, Hubei Province 430062, PR China

## Abstract

**Background:**

RNA interference (RNAi) has become a powerful means for silencing target gene expression in mammalian cells and is envisioned to be useful in therapeutic approaches to human disease. In recent years, high-throughput, genome-wide screening of siRNA/miRNA libraries has emerged as a desirable approach. Current methods for constructing siRNA/miRNA expression vectors require the synthesis of long oligonucleotides, which is costly and suffers from mutation problems.

**Results:**

Here we report an ingenious method to solve traditional problems associated with construction of siRNA/miRNA expression vectors. We synthesized shorter primers (< 50 nucleotides) to generate a linear expression structure by PCR. The PCR products were directly transformed into chemically competent *E. coli *and converted to functional vectors *in vivo *via homologous recombination. The positive clones could be easily screened under UV light. Using this method we successfully constructed over 500 functional siRNA/miRNA expression vectors. Sequencing of the vectors confirmed a high accuracy rate.

**Conclusion:**

This novel, convenient, low-cost and highly efficient approach may be useful for high-throughput assays of RNAi libraries.

## Background

Rapid gene silencing by means of RNA interference (RNAi) has become a powerful way of studying the function of genes on a genome-scale in cultured mammalian cells [1-3]. High-throughput assays of RNAi libraries have facilitated the search for genes required for diverse biological processes, thus allowing stepwise dissection of specific signaling pathways and of the regulation of gene function [4]. RNAi has been applied to genome-wide reverse genetics in *Caenorhabditis elegans *[5]. The problem of long (> 30 nucleotides, nt) double-stranded RNAs (dsRNAs) eliciting interferon responses in higher vertebrates has been conquered by the finding of short (21–23 nt) dsRNAs [6]. The RNAi response in mammalian cells mediated by dsRNA is a well-known two-step process [7,8]. Initially, the dsRNA is cleaved by an RNase III-like enzyme known as Dicer, which processes dsRNA into ~22-nt small interfering RNAs (siRNAs). Then the duplex siRNAs are passed to the RNA-induced silencing complex (RISC), which is activated by unwinding of the duplex. Activated RISC complexes can regulate gene expression at many levels [9,10].

Several approaches can be used to induce gene silencing. The chemical synthesis method is commonly used, but it is very expensive [11]. The limited capacity of *in vitro *transcribed siRNA and unknown available siRNA digested by long dsRNA (200–1000 bp with the T7 promoter) with RNase III/Dicer limit the application of these methods [12-15]. An alternative approach that has attracted considerable interest is the generation of short hairpin RNA (shRNA) by transfecting a plasmid or transducing a viral vector encoding an shRNA driven by an RNA polymerase III promoter (U6, H1, 7SK and tRNA promoters) or polymerase II promoter (CMV or SP-C) [16-21]. shRNA, which consists of short inverted repeats linked by a small loop sequence, can be processed into 19–22-nt siRNA in cells that may suppress expression of the target gene [22,23]. Studies have demonstrated that the gene silencing effect induced by transfection of shRNA expression vectors is comparable to that induced by chemically synthesized siRNA. Furthermore, several advantages of shRNA expression vectors mean that this approach is very suitable for inducing gene silencing. First, the method is fairly inexpensive. It also solves the problem of long-term target gene suppression in cells and whole organisms. Finally, shRNA expression cassettes of vectors can be stably integrated into the host genome using viral systems [23,24].

A typical strategy for the construction of shRNA expression vectors in mammalian cells requires synthesis, annealing and ligation of two long complementary oligonucleotides consisting of the whole shRNA with a terminal signal of 5–6 nt and extra nucleotides for cloning [25]. This method suffers from mutation of long oligonucleotides, and synthesis and purification of both oligonucleotides are costly. Many other methods can be used to reduce the mutation problem, but most of them require at least one long oligonucleotide, which is costly, or special processing [26-30].

Here we report an efficient and inexpensive method for the generation of shRNA expression vectors for *in vitro *transcription that is based on one-step PCR and homologous recombination. Recently, primers with only 9 nt complementary to the template have been subjected to PCR and the products or plasmid fragments with homologous regions can subsequently automatically recombine *in vivo *[31]. Therefore, it should be possible to construct shRNA expression vectors by transforming PCR products into chemically competent cells, using a convenient procedure to screen for positive clones under UV light [32,33]. The approach can be used to produce many different shRNA expression vectors. Using this method, we have constructed 442 shRNA expression vectors, most of which were correctly sequenced. We have also constructed 119 microRNA expression vectors using the same approach. Our results demonstrate that this is a useful approach for constructing siRNA/miRNA expression vectors at a greatly reduced cost with high efficiency and has potential for establishing siRNA/miRNA libraries.

## Results

### Construction of negative control vectors

**Figure 1:**
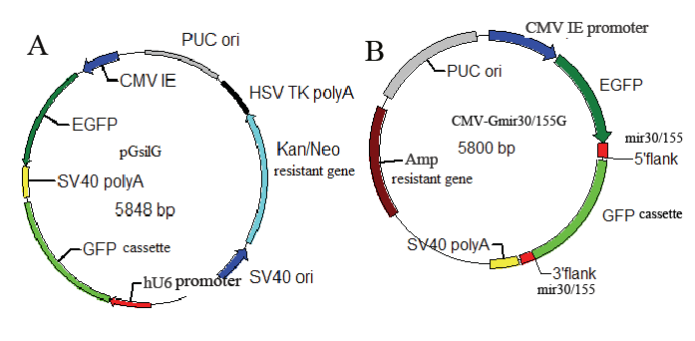
**Illustrations of the features of pGsilG (A) and CMV-Gmir30/155G (B)**. pGsilG is constructed based on the shRNA expression vector pGenesil1.0. A GFP cassette is inserted between hU6 promoter and SV40 polyA. CMV-Gmir30/155G is constructed based on T-vector. A miRNA expression cassette (CMV IE-EGFP-mir30/155 5' flank-GFP cassette-mir30/155 3' flank-SV40 polyA) is inserted in MCS. EGFP driven by CMV IE promoter is fused with GFP cassette and this feature will turn to the result of EGFP-miRNA fusion structure.

**Figure 2:**
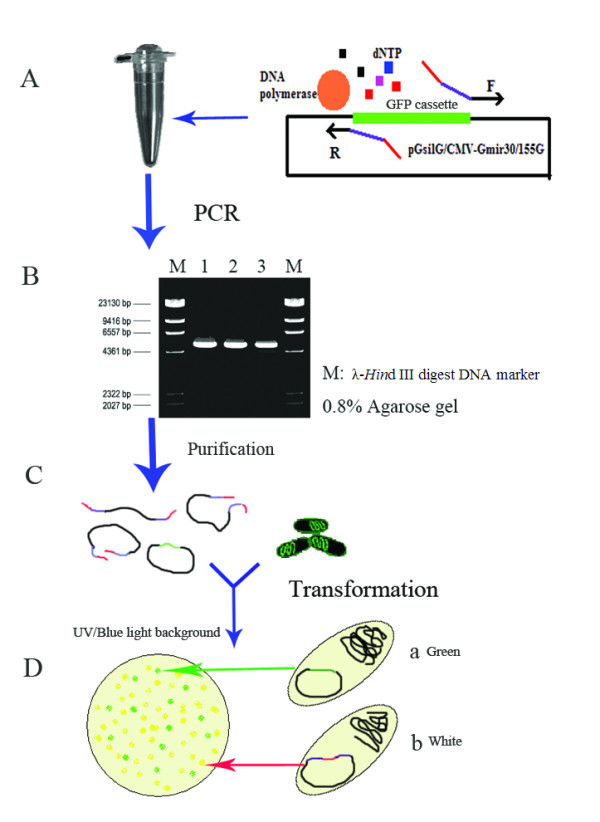
**Main procedures for the one-step PCR process**. First, an appropriate PCR system and program should be adopted (A). Then the PCR products need to be authenticated and purified (B). After testing and purification, the PCR products can be transformed into chemically competent *E. coli *(C). Finally, positive clones (Db) are easily selected because negative clones (Da) exhibit green fluorescence under UV light.

**Figure 3:**
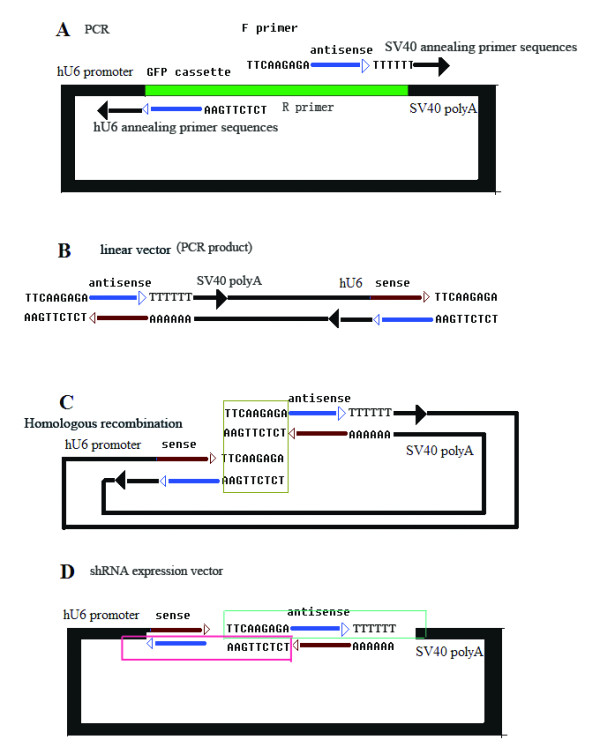
**Schematic diagram of the one-step PCR process**. (D) shows the target structure: hU6 promoter-sense-loop-anti-sense-TTTTTT-SV40 polyA. The two panes indicate the primer sequences. The primer sequences are shown in (A). PCR then yields the linear vector shown in (B). It is obvious that a homologous region on each side, as shown in (C). Product transformation into chemically competent *E. coli *cells yields the structure shown in (D).

pGsilG (Figure 1A) and CMV-Gmir30/155G (Figure 1B) were constructed based on pGenesil1.0 (Genesil Biotechnology Co. Ltd.) and the T-vector (TaKaRa), respectively. The vectors were used as negative controls for screening shRNA/miRNA expression vectors in *E. coli *and for cell transfection. EGFP driven by eukaryotic promoter CMV IE can express green fluorescence in HEK293T or HeLa cells but not express in *E. Coli*. The green fluorescence protein (GFP) cassette can be expressed in *E. coli *and clones exhibit green fluorescence under UV light, so that it is easy to screen positive clones. Since the GFP cassette is not present in the PCR product sequence, after product transformation into competent *E. coli *cells positive clones are white and negative clones exhibit green fluorescence under UV light (Figures 2 and 3). In our experiments two or three positive clones were usually selected for automated sequencing.

The pGsilG plasmid has a hU6 promoter that drives shRNA expression and a CMV IE promoter that drives EGFP expression in eukaryotic cells. The CMV-Gmir30/155G plasmid has a CMV IE promoter that drives both miRNA and EGFP expression in eukaryotic cells. EGFP expression provides a visual indication of the transfection efficiency.

### Construction of shRNA/miRNA expression vectors

Primer design is extremely important for PCR (Figure 3A). The primer sequences should be as follows: forward primer, 5'-loop region-anti-sense-TTTTTT-SV40 annealing region-3'; reverse primer, 5'-homologous region (complementary to loop) -anti-sense-hU6 annealing region-3'. Here 9-nt loop sequences are adopted as the homologous region. The annealing temperature depends on the length of the hU6 and SV40 annealing primer sequences. In the present study, 11–15 nt length of the hU6 or SV40 annealing primer sequences and an appropriate annealing temperature were chosen. The primers' 5' loop regions need at least 9 nt complementary to each other, which is sufficient for *in vivo *recombination in chemically competent *E. coli *(> 1 × 10^8 ^cfu/μg DNA).

The PCR program is also crucial for successful experiments. In our experience, the PCR conditions are relatively standard for all shRNA/miRNA genes because the regions complementary to the template do not change. In the present study, the conditions for KOD-Plus *Taq *polymerase were as follows: 15 s at 94°C, 30 s at 38°C and 5 min at 68°C for 25 cycles. *Pfu *DNA polymerase was occasionally used with conditions as follows: 30 s at 95°C, 30 s at 40°C and 5 min at 72°C for 25 cycles.

All the PCR products should be authenticated and then purified. Gel extraction can reduce the template background. Alternatively, the solution reclaiming method is also suitable, for which Montage™ PCR centrifugal filter devices (Millipore) and ethanol precipitation were used in the present study. Then purified PCR products were transformed into chemically competent *E. coli *cells directly. All of the steps were completed within 8 h. The mechanism for recombination of PCR products *in vivo *is not clear, but we assumed that this occurs in line with homologous recombination theory and that the cell repair system plays an important role.

### Sequencing of positive clones

In our research, two or three positive clones were usually randomly selected for automated sequencing. The accuracy was satisfactory: only 55 out of 442 shRNA and 18 out of 119 miRNA expression plasmids contained a mutation. These results indicate that our method is less error-prone that common approaches, which have a mutation rate of 25–50%.

### RT-PCR and Western blotting

**Figure 4:**
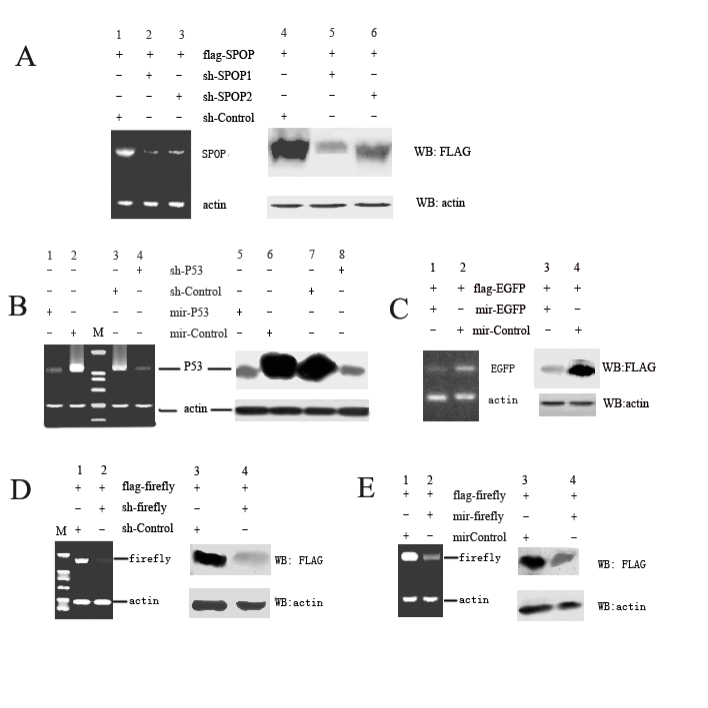
**RT-PCR (each *left panel*) and Western blot (each *right panel*) results**. (A) HEK293T cells were transfected with a vector encoding flag-SPOP and SPOP-specific shRNA vectors (shSPOP1 and shSPOP2) or a negative control vector (shControl). After incubation for 48 h, total RNAs were prepared and subjected to reverse transcription-PCR for SPOP and actin mRNAs. Cell lysates were subjected to western blot with anti-FLAG antibody. (B) HEK293T cells were transfected with p53-specific shRNA/miRNA vectors (shP53/mirP53) or a negative control vector (shControl/mirControl). Anti-p53 antibody was used. (C) demonstrates the RT-PCR and western blot results for EGFP gene silencing. (D) and (E) demonstrate the RT-PCR and western blot results for firefly luciferase gene silencing.

We present some representative RT-PCR and Western blot results for SPOP (*Homo sapiens *speckle-type POZ protein, GI:56119172), EGFP (Enhanced Green Fluorescence protein), p53 (*Homo sapiens *tumor protein p53, GI:120407067) and Firefly luciferase (*Photinus pyralis *firefly luciferase, GI:197215837) RNAi in Figure 4. The targets selected are shown in table 1 and the primers used in RT-PCR are listed in table 2. We constructed these shRNA/miRNA expression vectors using one-step PCR and tested their interference ability in HEK293T cells. The results in Figure 4 demonstrate that these expression vectors are efficacious. The plasmids flag-SPOP, flag-EGFP and flag-firefly combine the SPOP, EGFP and firefly luciferase genes with the FLAG tag so that these proteins can be detected using an anti-Flag antibody.

**Table 1:** The RNAi targets used in this paper.

Name	Target
shSPOP1	5' TTCCAGGCTCACAAGGCTATC 3'

shSPOP2	5' CTATCATGCTTCGGATGTC 3'

shP53	5' GACTCCAGTGGTAATCTAC 3'

mirP53	5' AGACTCCAGTGGTAATCTA 3'

mir-EGFP	5' GGCGATGCCACCTACGGCAAG 3'

sh-firefly	5' AAGCGCTATGGGCTGAATACA 3'

mir-firefly	5' AATACAAACCATCGGATCGTG 3'

**Table 2:** The primers used in RT-PCR.

SPOP-F	5' GTCCTCCACCTCCGGCAGAA 3'
SPOP-R	5' GGATTGCTTCAGGCGTTTGC 3'

P53-F	5' ATGGAGGAGCCGCAGTCAGA 3'

P53-R	5' GTCTGAGTCAGGCCCTTCTGTCTT 3'

EGFP-F	5' ATGGTGAGCAAGGGCGAGGA 3'

EGFP-R	5' TTACTTGTACAGCTCGTCCATGCCG 3'

FIRE-F	5' GCCCAGCGCCATTCTACCCACTCG 3'

FIRE-R	5' TGCCGCCCTTCTTGGCCTTAATG 3'

actin-F	5' GGGAGAGCGGGAAATCGTGCGTGA 3'

actin-R	5' GATGGAGTTGAAGGTAGTTTCGTG 3'

## Discussion

A GFP cassette was inserted into the normal shRNA expression vector pGenesil1.0 between the hU6 promoter and SV40 polyA. A CMV-Gmir30/155G vector was also constructed with the GFP cassette between the CMV promoter and SV40 polyA. The GFP cassette can be expressed in prokaryotic cells such as *E. coli *because it has a prokaryotic promoter itself and is thus an outstanding screening label, making it much simpler to screen recombinants. The cassette is eliminated after the PCR process and thus recombinants do not exhibit green fluorescence under UV light after product transformation into *E. coli *cells. Positive clones can then be directly selected for sequencing.

Since the whole vector is reproduced in the PCR procedure, DNA polymerase of high quality and fidelity should be used. Use of poor *Taq*/DNA polymerase can result in unsatisfactory transfection in spite of the correct shRNA sequences, because eukaryotic expression components might mutate during PCR. Thus, KOD-Plus *Taq *polymerase (Toyobo) and *Pfu *DNA polymerase (Promega) were used in our experiments and yielded satisfactory results. The error rates of these polymerases are approximately 1.3 × 10^-6 ^per bp duplicated. Therefore, under the condition of 25 amplification cycles, approximately 1.1% of the products would include mutations in the hU6 hairpin region and 2.2% of the products may include mutations in the CMV hairpin region. These low-probability events would not affect the transfection experiment. In this method, since the whole vector is amplified by PCR, there is a possibility that some mutations may be inserted somewhere in the vector, even if using high-fidelity enzyme. But in our research, no such mutations caused by this possibility have been found. The problem could be avoided by performing a homology search: the loop and target sequences should not be homologous with the vector skeleton. Furthermore, the primers' annealing regions which are complementary to the template are predesigned and unchanged when using the same vector skeleton so that the problem rarely happens. For example, when using pGsilG as PCR model, the forward primer's annealing region is *TTT TTT CCC GGG ACG *and the reverse primer's annealing region is *GGA TCC CGC GTC C*.

Primer quality is associated with sequence accuracy. All primers used should be purified by PAGE. The 3' sequences affect annealing during PCR and the 5' sequences are related to recombination *in vivo*, so mutation of these sites will lead to failure, with no PCR products or no positive clones obtained. We observed that mutations nearly always occurred in target insertion and deletion sequences, probably during primer synthesis. When the quality of primers was assured, fewer mutations occurred.

The recombination of PCR products *in vivo *may cause mutations of the shRNA region. The mutation rates of *E. coli *are known to be approximately 10^-6^, but empirical evidence suggests that mutation rates in palindromic regions of plasmids are high. This indicates that mutations in the shRNA region are associated with the shRNA sequence, so the target selected should follow the Tuschl rules and avoid repeats or complex sequences. In particular, the shRNA region is separated into two parts at the end of the PCR product, which would reduce mutations during recombination *in vivo*.

Since the screening of effective RNAi targets always requires the preparation of more than three different shRNA expression vectors, it is costly and time-consuming to use normal methods when performing high-throughput assays. The normal method also suffers from mutation problems. Typically, 25–50% of cloned shRNA constructs contain significant mutations as determined by DNA sequencing. The mutation frequency is close to 75% when the desired siRNA sequence is 29 nt in size. The unreliability is in part due to errors for long oligonucleotides of > 50 nt. The two complementary oligonucleotides encoding a desired shRNA target sequence are almost 60 nt in length. For instance, oligonucleotides encoding 19- and 29-nt target sequences with extra enzyme-cutting sites are 57 and 77 nt, respectively. These oligonucleotides are hard to synthesize and are unreliable. However, in our approach the primer length is < 45 nt so that the accuracy of positive clones is dramatically improved. Even for miRNA expression, the primer length can be controlled to < 48 nt in size. This design of short primers is extremely useful for overcoming mutation problems and it is very inexpensive and less time-consuming.

## Conclusion

The method proposed not only reduces costs, but is also more efficient since no digestion or ligation procedures are required. The entire process for formation of a linear vector can be completed within a PCR tube, and the recombinant clones are easily screened after transformation of the PCR products. We demonstrated an alternative approach for construction of shRNA/miRNA vectors that is inexpensive and highly efficient. Cost savings could be as much as 50% for primer synthesis to DNA sequencing. First, linear products that can be converted to siRNA/miRNA expression vectors in competent *E. coli *can be generated using a one-step PCR process. Second, the design of short primers greatly decreases mutation problems and costs. Finally, no restriction endonuclease digestion or DNA ligation is required, meaning that this approach can save time and resources. Thus, this highly efficient and reliable approach may be useful for high-throughput or genome-wide screening of RNAi libraries.

## Methods

### Construction of negative control vectors by molecular cloning

We reconstructed pGenesil1.0 (Genesil Biotechnology Co. Ltd.) by inserting a GFP cassette, which can be expressed in prokaryotic cells, between the hU6 promoter and SV40 polyA. The pGenesil1.0 plasmid and GFP cassette were digested using the restriction endonucleases *Hin*d III (TaKaRa) and *Bam*H I (TaKaRa) and then ligated with T4 DNA ligase (TaKaRa).

We also constructed the CMV-Gmir30/155G vector based on the T-vector (TaKaRa) by molecular cloning. The CMV IE promoter, EGFP, the mir30/155 flanking region, a GFP cassette and SV40 polyA were inserted in MCS (Multiple Cloning Sites) of the T-vector.

### Construction of shRNA expression vectors

The following steps were used to construct a human U6 promoter-driven shRNA vector with a recommended loop sequence of 5'-***TTCAAGAGA***-3' as proof of principle.

#### Step 1: Primer design

Two short primers were designed for PCR with the template pGsilG. The sequence of the forward primer was 5'-***TTC AAG AGA ***N_(19–23)_TTT TTT CCC GGG ACG-3' and that of the reverse primer was 5'-***TCT CTT GAA ***N_(19–23)_GGA TCC CGC GTC C-3'. The 3' annealing primer sequences, which are complementary to the template, could be shorter than that recommended for a lower annealing temperature. The 5' loop sequences are necessary for recombination *in vivo*. For instance, if the target sequence is 5'-CCA CAC AAC CTG GTA GCA T-3', the shRNA structure should be of the form 5'-CCA CAC AAC CTG GTA GCA T***TT CAA GAG A***AT GCT ACC AGG TTG TGT GGT TTT TT-3'.

Thus, the sequence of the forward primer should be 5'-***TTC AAG AGA ***ATG CTA CCA GGT TGT GTG GTT TTT TCC CGG GAC G-3' and that of the reverse primer should be 5'-***TCT CTT GAA ***ATG CTA CCA GGT TGT GTG GGG ATC CCG CGT CC-3'.

All primers were ordered as general standards and were purified by PAGE and re-suspended in TE buffer (10 mM Tris, pH 8.0, 1 mM EDTA) at a concentration of 20 pM/μL. The short length of the primers (≤ 45 nt) ensures their quality.

#### Step 2 PCR process

To obtain abundant and reliable products, high-quality and high-fidelity DNA polymerase was used. In this experiment, KOD-Plus *Taq *polymerase (Toyobo) or *Pfu *DNA polymerase (Promega) was used for PCR in a volume of 50 μL, with annealing at 38°C.

#### Step 3 Purification of PCR products and transformation

After authentication, the PCR products were purified using a Montage™ PCR centrifugal filter device (Millipore). Then the purified PCR products were transformed into chemically competent *E. coli*. The transformed cells were spread on Luria-Bertani agar plates containing antibiotics and incubated at 37°C overnight. Positive clones were selected under UV light and confirmed by automated sequencing using the hU6 sequencing primer (5'-CCA AGG TCG GGC AGG AAG AG-3').

### Construction of miRNA expression vectors

We constructed mir30/155 miRNA expression vectors in a parallel design. The difference depends on the primer design. We used the internet design system BLICK™-IT RNAi Designer (Invitrogen) to select appropriate targets. In the case of mir30, for example, the loop sequence is 5'-***TAG TGA AGC CAC AGA TGT A***-3' to obtain a structure such as: 5'-mir30flank-(G)TAA TCC TGA AGG CTC CTC AGA ***TAG TGA AGC CAC AGA TGT A***TC TGA GGA CTT CAG GAT TA-mir30flank-3'. The forward primer was 5'-***AAG CCA CAG ATG TA***T CTG AGG ACT TCA GGA TTA *TGC CTA CTG CC*-3'. The reverse primer was 5'-***CTG TGG CTT CAC TA***T CTG AGG AGC CTT CAG GAT TA*C GCT CAC TGT CAA*-3'.

Here we separated the loop sequence into two parts with 9 nt complementary to each other for recombination *in vivo*.

### Cell culture and transfection

HeLa and HEK293T cells were grown in DMEM supplemented with 10% inactive fetal calf serum (FBS), 1 mM L-glutamine, and 100 U/mL streptomycin, and incubated at 37°C in 5% CO_2_.

One day before transfection, cells were plated at a density recommended by the manufacturer of the transfection reagent (FuGENE^® ^HD). The target expression plasmid and shRNA/miRNA expression plasmid were transfected into cells according to the manufacturer's instructions. The target expression plasmid and negative control plasmid were transfected as a negative control. Separate wells were used for separate constructs. The medium was removed and replaced with fresh growth medium 4–6 h after transfection. The cells were incubated overnight at 37°C and harvested the next day to determine the efficacy of silencing of the target gene.

### RT-PCR analysis

RT-PCR was used to semi-quantify mRNA expression level of genes. Briefly, total RNAs were isolated from cells using Trizol agent (Invitrogen). Reverse transcription reactions were performed using Superscript III reverse transcriptase (Invitrogen) and oligo(dT) primer by following the manufacturer's instruction. The mRNAs were amplified with primers listed in table 2. As internal standard, a fragment of human endogenous β-actin was amplified simultaneously in each PCR reaction. PCR products were resolved on a 2.0% agarose gel and the bands were visualized by ethidium bromide staining.

### Western blot analysis

The cells were harvested and lysed with lysis buffer (250 mM sucrose, 1% Triton X-100, 2 mM EDTA, 10 mM EGTA, 50 mM Tris-HCl, pH 7.4, 200 μg/mL leupeptin). The cell lysate (10–20 μg of protein) was separated by SDS-PAGE and electrophoretically transferred onto a nitrocellulose membrane (Immobion™ transfer membrane, Millipore, USA). After blocking non-specific binding sites with 5% non-fat milk, the membrane was incubated with primary antibodies for 1 h at room temperature. The primary antibodies used were anti-Flag (Sigma, 1:2000 dilution), monoclonal anti-p53 antibody (Sigma,1:1000 dilution) and anti-actin antibody (Sigma, 1:2000 dilution). After washing, the blot was incubated with goat anti-mouse IgG (H+L)-HRP, and immunoreactive bands were visualized using a Super signal maximum sensitivity kit (Thermo Scientific).

## Authors' contributions

JX designed the experiments, carried out the practical work and drafted the manuscript. LXM proposed the original idea, supervised all the studies and helped to finalize the manuscript. JQZ assisted JX in construction of shRNA expression vectors. GW identified siRNA sequences that are capable of silencing SPOP. GBH assisted JX in cell culture. HY helped to amend the manuscript. All authors read and approved the final manuscript.
